# A Preliminary Cadaveric MRI Study of Fetal Hip Development

**DOI:** 10.3389/fsurg.2022.847135

**Published:** 2022-02-14

**Authors:** Zhenqing Liu, Huixian Li, Shuai Wang, Qianqian Wu, Hongsheng Liu

**Affiliations:** ^1^Department of Radiology, Guangzhou Woman and Children's Medical Center, Guangzhou Medical University, Guangzhou, China; ^2^Institute of Pediatrics, Guangzhou Woman and Children's Medical Center, Guangzhou Medical University, Guangzhou, China

**Keywords:** acetabular index, development, fetal hip, MRI, MULTTEST

## Abstract

**Purpose:**

The earlier the detection of the hip joint is discovered, the better the final result. The purpose of this study aimed to investigate the fetal hip development using magnetic resonance imaging (MRI), so as to alert clinicians to possible abnormal development during intrauterine life.

**Method:**

Measurements of 34 cadaver fetuses (68 hips) were obtained regarding acetabular width and depth, anterior bony acetabular index (ABAI), anterior cartilaginous acetabular index (ACAI), posterior bony acetabular index (PBAI), and posterior cartilaginous acetabular index (PCAI). The standard values of each acetabular measurement index were obtained, and the gestational age-measurement index change trend chart was drawn to comprehensively analyze the normal development law of the fetal hip joint.

**Results:**

With the development of fetuses, the width and depth of acetabular increase linearly, and the slope of acetabular width was larger than that of depth. In addition, two change points during the 24th and 34th weeks of gestation were detected with regard to width. ABAI and PBAI also decreased. ABAI demonstrated an approximately linear trend, while PBAI shows a non-linear trend. During the 36th week, the change point in PBAI was observed. ACAI and PCAI exhibited slow increases, indicating a non-linear trend. During the 21st and 36th weeks of gestation, the change points regarding ACAI were observed. During the 22nd week of gestation, the change point for PCAI was observed.

**Conclusion:**

Plots of the parameters obtained *via* MRI examinations of cadaver fetuses across gestational age comprehensively illustrated the fetal hip development. This developmental information about the hip joint has the potential to guide clinicians in the early detection of abnormal hip joint development during intrauterine life.

## Introduction

Developmental dysplasia of the hip (DDH) is a common teratogenesis disease in children. The risk factors for DDH are breech presentation, family history, and oligohydramnios (1). Previous studies have shown that late DDH usually leads to more complicated treatments and increased long-term complications (2). The earlier DDH is discovered, the better the final result will be (3). Although high-risk DDH infants are usually formally examined at birth, it is not possible to find dislocation of hip joint until the children are obviously limping. This is because clinical symptoms are not obvious in the early childhood (2). There is evidence that abnormal mechanical forces acting on the hip joint in uterus is related to the occurrence of DDH disease (1, 4). Investigators pay more attention to indicate the prenatal hip development (5). Published research shows that this information is usually gained from post-mortem examinations or ultrasonic imaging (6–8).

And the shape of acetabulum and femoral head have only been monitored in anatomical studies (6, 7). Also, various indicators have been used in ultrasonographic studies to assess the fetal hip development (8). Graf described the most extensive method, which is implemented by measuring the acetabular obliquity (the α angle) and the acetabular cartilage apex angle (the β angle) (9). Baróti et al. concluded that the hip joint was less stable during perinatal life, as the α angle decreased (8). In DDH, the left hip is more common, because it is adducted against the mother's lumbosacral spine in the most common intrauterine position (10). However, the left hip was a poor show on ultrasound in the case of breech presentation (11).

In 2007, Whitby et al. indicated magnetic resonance imaging (MRI) as a valid method for assessing fetal hip development owing to its high resolution and satisfactory soft tissue contrast (12). Measure the width and depth of acetabulum, the radius and diameter of femoral head, volume and area, and fully reflect the shape of femoral head. However, insufficient coverage of the femoral head is a significant precursor of a hip dysplasia (13, 14).

According to the literature (15, 16) and the accumulated experience, specific parameters were measured in this study using MRI obtained from 34 cadaver fetuses (68 hips) ranging in age from 18 to 42 weeks of gestation, so as to comprehensively evaluate coverage of the femoral head of fetal hip joint. Acetabular width and depth were measured to indicate the morphology of the acetabulum, and the anterior bony acetabular index (ABAI), anterior cartilaginous acetabular index (ACAI), posterior bony acetabular index (PBAI), and posterior cartilaginous ace-tabular index (PCAI) were measured to indicate the anterior or posterior acetabular coverage of the femoral head.

The parameters obtained using MRI of cadaveric fetuses of different gestational ages were plotted to investigate the development of fetal hip, and to remind clinicians to find abnormal hip development early during intrauterine life, so as to assess pathological severity and make emergency interventions.

## Materials and Methods

### Fetal Specimens

The study was approved by the hospital ethics committee. Informed consent was obtained from the individuals concerned for the storage and use of the fetus for study purposes. The 68 hip joints of 34 cadaver fetuses (aged 18–42 weeks of gestation; nine females and 25 males) were included from 2012 through 2016 to investigate the development of fetal hip. Fetuses older than 18 weeks were required because the ossification of the acetabulum started by that time (8). The all fetuses were spontaneous miscarriages in the hospital with the exception of congenital malformations affecting fetal hip development, validated by ultrasound, absence of maternal drug use, breech position, twin fetuses, and family history of DDH. And the fetuses were excluded when gestational age was inconformity with the actual assessing both last menstruation date and ultrasonography. None of the fetuses were from a vulnerable population and all donors or next of kin provided written informed consent that was freely given.

### Experimental Grouping

All the subjects were divided into five groups according to different gestational ages: 18–22 weeks, eight cases, 16 hips in total; 23–27 weeks, five cases, 10 hips in total; 28 to 32 weeks, seven cases, 14 hips in total; 33 to 37 weeks, seven cases, 14 hips in total; 38–42 weeks, seven cases, a total of 14 hips.

### Imaging Methods

The hips were scanned using 1.5T MRI (Philips, Achieva, The Netherlands) within 24 h of death. Take the fetus in supine position, straighten the lower limbs together, moderately press the calf and bilateral hip joints with sandbags, and place the midpoint of the connecting line of bilateral hip joints at the center of the coil. T2-weighted images (T2WIs) were obtained using eight-channel SENCE cardiac coils in T2-weighted fast spin-echo (repetition time 2,137 ms; echo time 92 ms; time of acquisition 2 min 36 s; seam thickness 2 mm; matrix 110 × 110) in the transverse plane. The remaining imaging parameters varied slightly based on the size of the fetus. The scanning methods are mentioned in Whitby's study (12). The scan took approximately 20 min.

### Measurements

**Figure 1:**
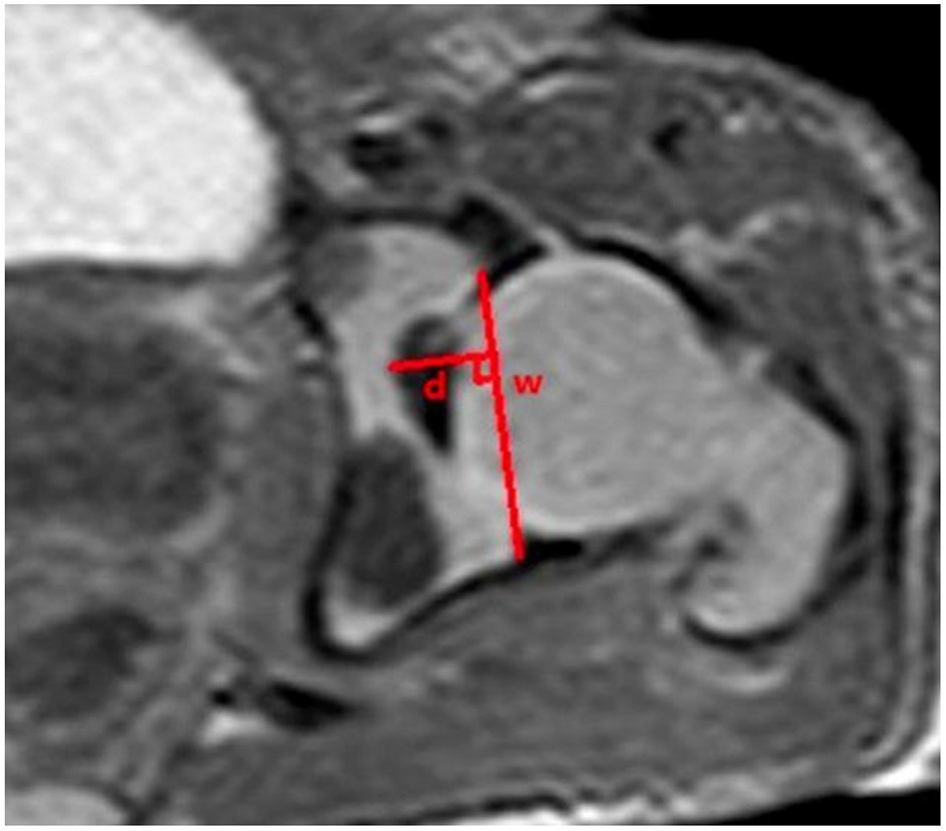
Distance w is the width of the acetabulum, and distance d is the depth of the acetabulum.

**Figure 2:**
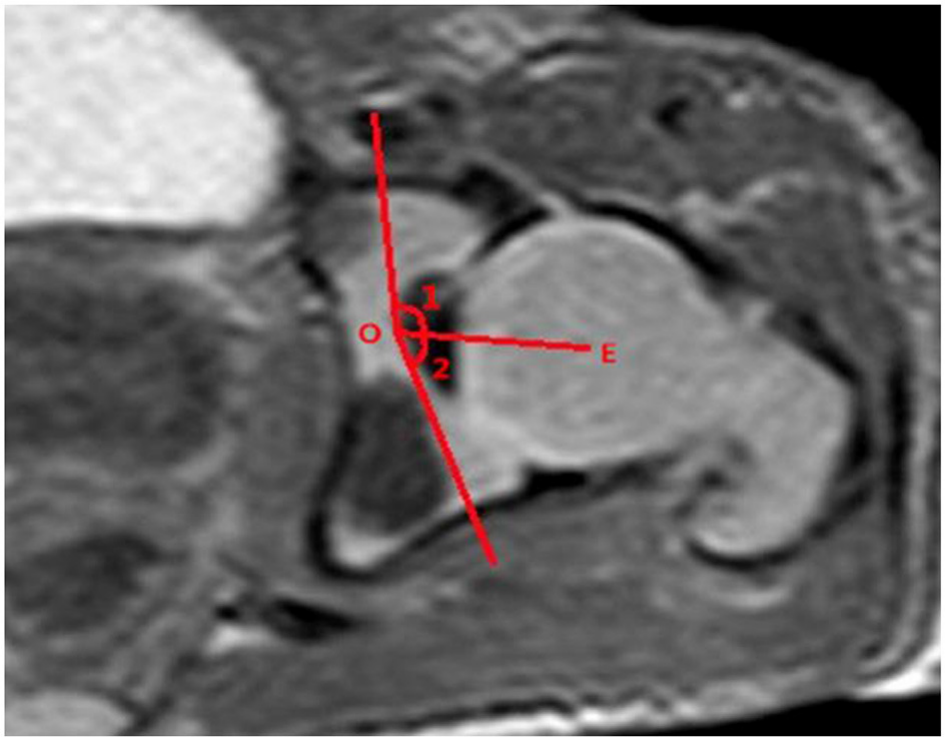
Line OE is the baseline. Angle 1 is the ABAI, and angle 2 is the PBAI.

**Figure 3:**
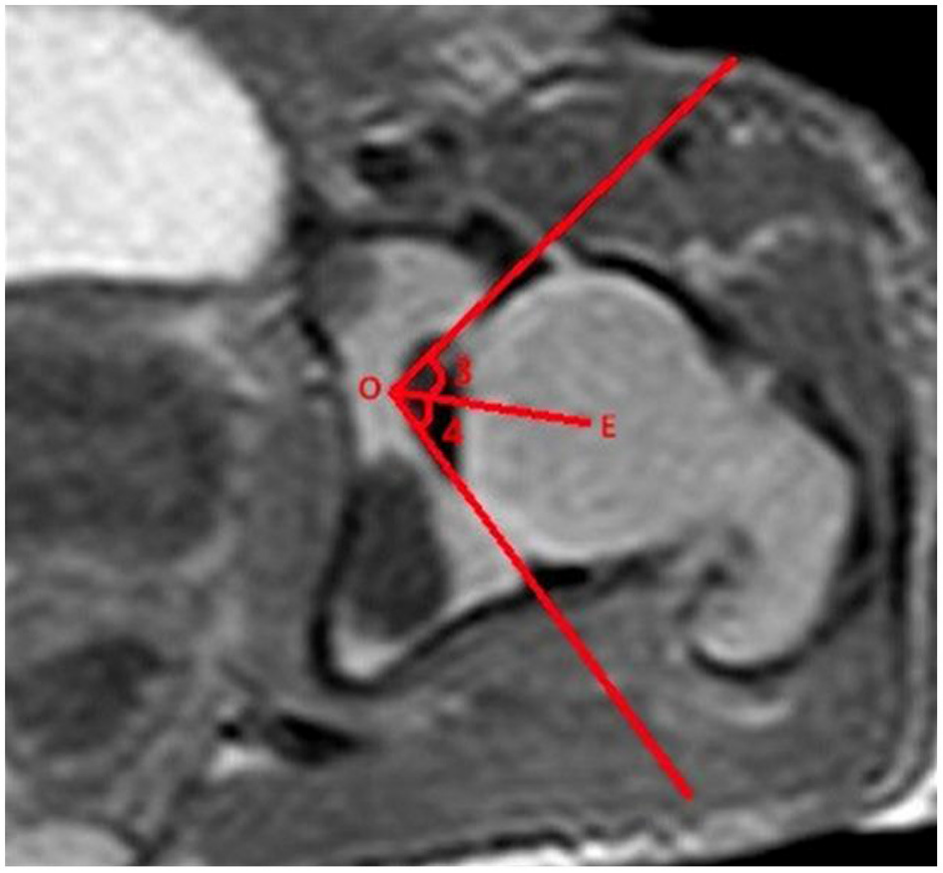
Line OE is the baseline. Angle 3 is the ACAI and angle 4 is the PCAI.

The measurements of acetabular width and depth, ABAI, ACAI, PBAI, and PCAI were completed using Picture Archiving and Communication System (PACS; YLZ, Fujian, China). The acetabular width was considered as the length of the line drawn from the anterior cartilaginous vertex to the posterior. The acetabular depth was considered as the perpendicular distance from the line to the maximum distance of the acetabulum (Figure 1). A line was drawn from the common center of the acetabulum and femoral head (point E) to the thinnest point of the acetabular cartilage (point O), which was regarded as the baseline. The baseline definition was similar to the method described by Harnroongroj et al. using computed tomography (16). The angles formed by the baseline and the line from the anterior or posterior osteal vertex of the acetabulum to point O represented ABAI and PBAI, respectively (Figure 2). The angles formed by the baseline and the line from the anterior or posterior cartilaginous vertex of the acetabulum to point O represented ACAI and PCAI, respectively (Figure 3). These angles were measured to represent the anterior or posterior acetabular coverage of the femoral head. Larger angles denoted less acetabular coverage of the femoral head. The obtained MR images of the hip joint were independently analyzed and measured by two senior radiologists who were in charge of imaging diagnosis, and each measurement was repeated three times. In the case of inconsistent measurement results, a third senior radiologists participated in the discussion and an agreement was finally reached.

### Statistical Methods

SPSS13.0 statistical software was used to establish the database and make statistical analysis. The sample (*n* = 68), which was used to collect data to draw the growth curve of the measurement, was divided into five age classes of comparable size according to the statistical standards. Moreover, comparisons were performed between the two sides and sexes. For each group, the means and the reference range of measurements were calculated. The fetal hip development was assessed in three steps. First, the MULTTEST procedure (SAS PROC MULTTEST) was performed to address multiple analyses and determine the trends for the six indicators. To correct for multiple comparisons, the *P*-value of each contrast was adjusted to a family-wise corrected α of 0.05 using Bootstrap permutation testing implemented in SAS 9.4 PROC MULTTEST (SAS Institute Inc., USA). Second, the normative trends of all parameters were plotted against gestational age to evaluate the relationships among the different age groups with regard to the indicators. Furthermore, linear or non-linear regression analyses were performed simultaneously with the corresponding R2 ≥ 0.60. Third, a change-point analysis (CPA) was used to detect the time point at which the statistical properties of a sequence of observations changed over time. Moreover, the CPA method was used to detect the changes in variability using the cpt.var function in R (version 3.2.5) (17). The detected change point(s) illustrated that the datasets could be divided into different segments with different variations, suggesting that the indicators changed across gestational age and at different rates. These change points are indicated in the corresponding regression graphs.

## Results

### Analysis of Consistency of Fetal Hip Joint Measurement Indexes in Different Gestational Age Groups

Six parameters were measured with regard to the 68 cadaver fetal hips ranging in age from 18 to 42 weeks of gestation to indicate the growth of the femoral head coverage during the fetal stage. All the inter-block correlation coefficients were close to 1, and the intra-class correlation coefficients were between 0.708 and 0.933 (*P* all < 0.05). No significant differences were observed between the two sides of the hip or between sexes (*P* all > 0.05). The means, reference ranges, and trends of the different groups are indicated in Table 1.

**Table 1:** Six indicators of femoral head coverage grouped by gestational age.

**GA**	**Num**	**Width (cm)**	**Depth (cm)**	**ABAI (°)**	**PBAI (°)**	**ACAI (°)**	**PCAI (°)**
18~	16	0.67 (0.40, 0.94)	0.30 (0.27, 0.34)	121.13 (110.39, 131.86)	73.99 (65.13, 82.85)	44.77 (38.60, 50.94)	43.76 (34.12, 53.40)
23~	10	0.91 (0.58, 1.24)	0.35 (0.23, 0.47)	105.22 (98.55, 111.88)	71.72 (67.07, 76.36)	50.30 (46.60, 54.00)	49.37 (44.35, 54.38)
28~	14	1.03 (0.82, 1.23)	0.39 (0.28, 0.50)	100.95 (82.18, 119.72)	70.76 (63.22, 78.30)	50.61 (45.88, 55.35)	51.26 (48.96, 53.57)
33~	14	1.34 (0.88, 1.79)	0.47 (0.31, 0.62)	94.55 (77.12, 111.97)	67.52 (53.98, 81.07)	51.05 (47.38, 54.72)	51.63 (49.69, 53.57)
38~	14	1.57 (1.39, 1.74)	0.56 (0.46, 0.66)	87.48 (81.43, 93.52)	56.48 (46.77, 66.18)	53.94 (46.97, 60.91)	52.43 (49.96, 54.89)
Pe^*^		<0.001	<0.001	<0.001	<0.001	<0.001	<0.001

*Characteristics are reported as means (95% confidence intervals). ABAI, anterior bony acetabular index; ACAI, anterior cartilaginous acetabular index; GA, gestational age; PBAI, posterior bony acetabular index; PCAI, posterior cartilaginous acetabular index. A total of 68 cases were divided into five groups of comparable size, and trends were observed with regard to all six indicators*;

* *P-values were adjusted to a family-wise corrected α of 0.05 using Bootstrap permutation testing implemented in SAS 9.4 PROC MULTTEST (SAS Institute Inc.)*.

### The Hip Joint Measurement Index and Change Trend of Gestational Age

**Figure 4:**
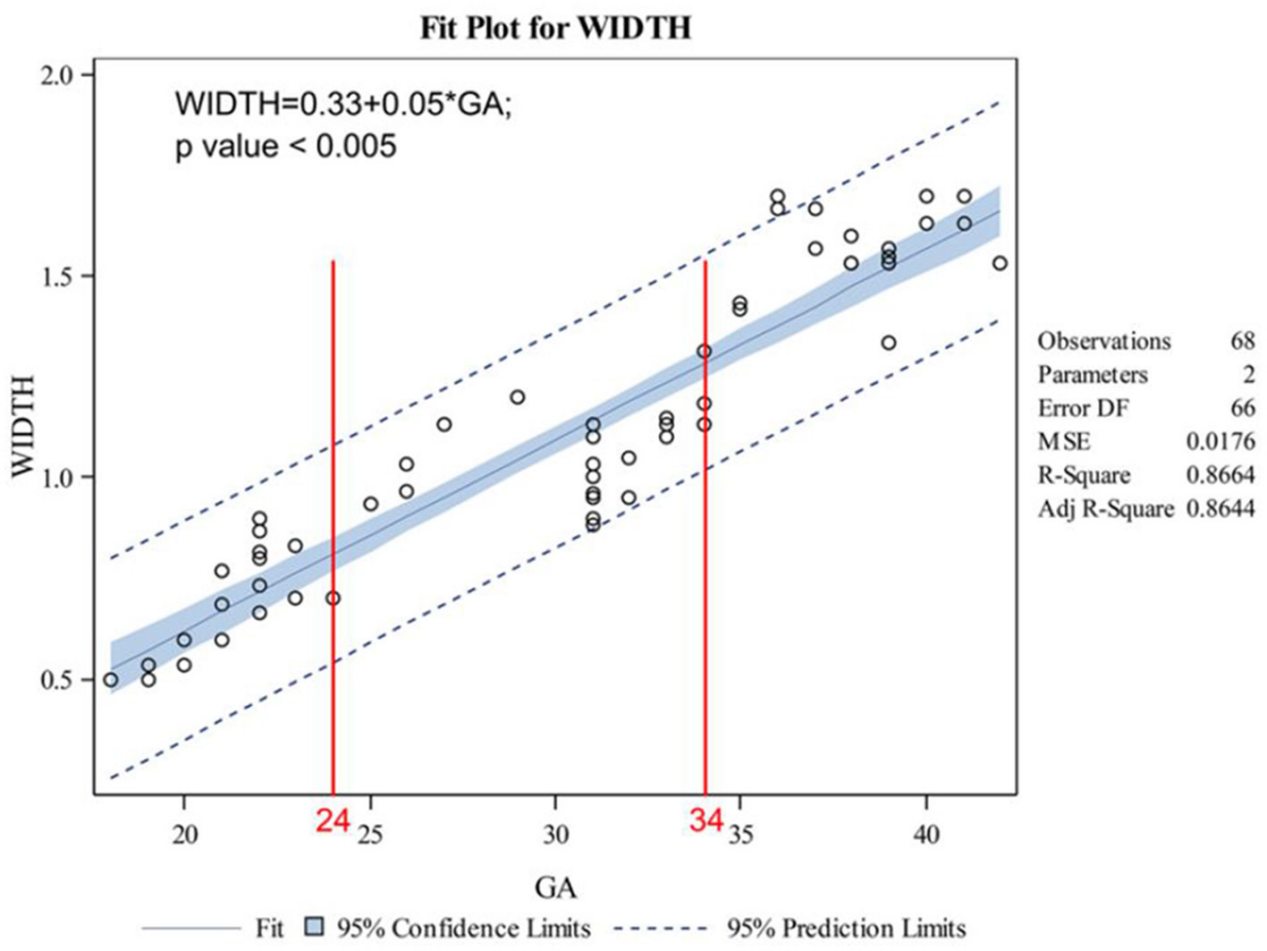
Correlation between gestational age and acetabular width.

**Figure 5:**
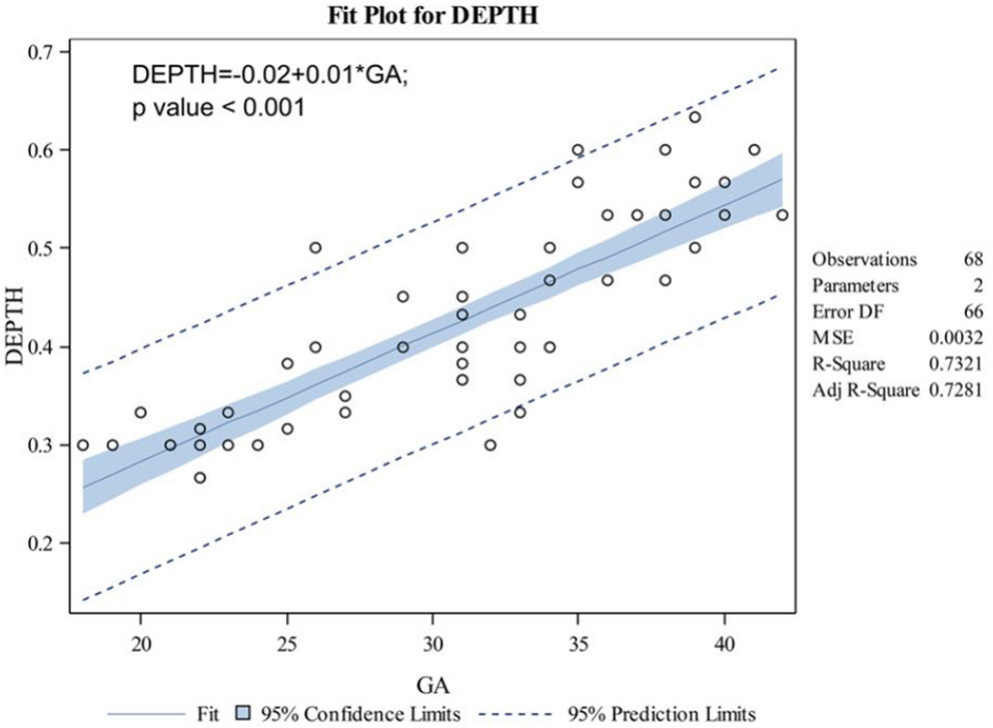
Correlation between gestational age and acetabular depth.

**Figure 6:**
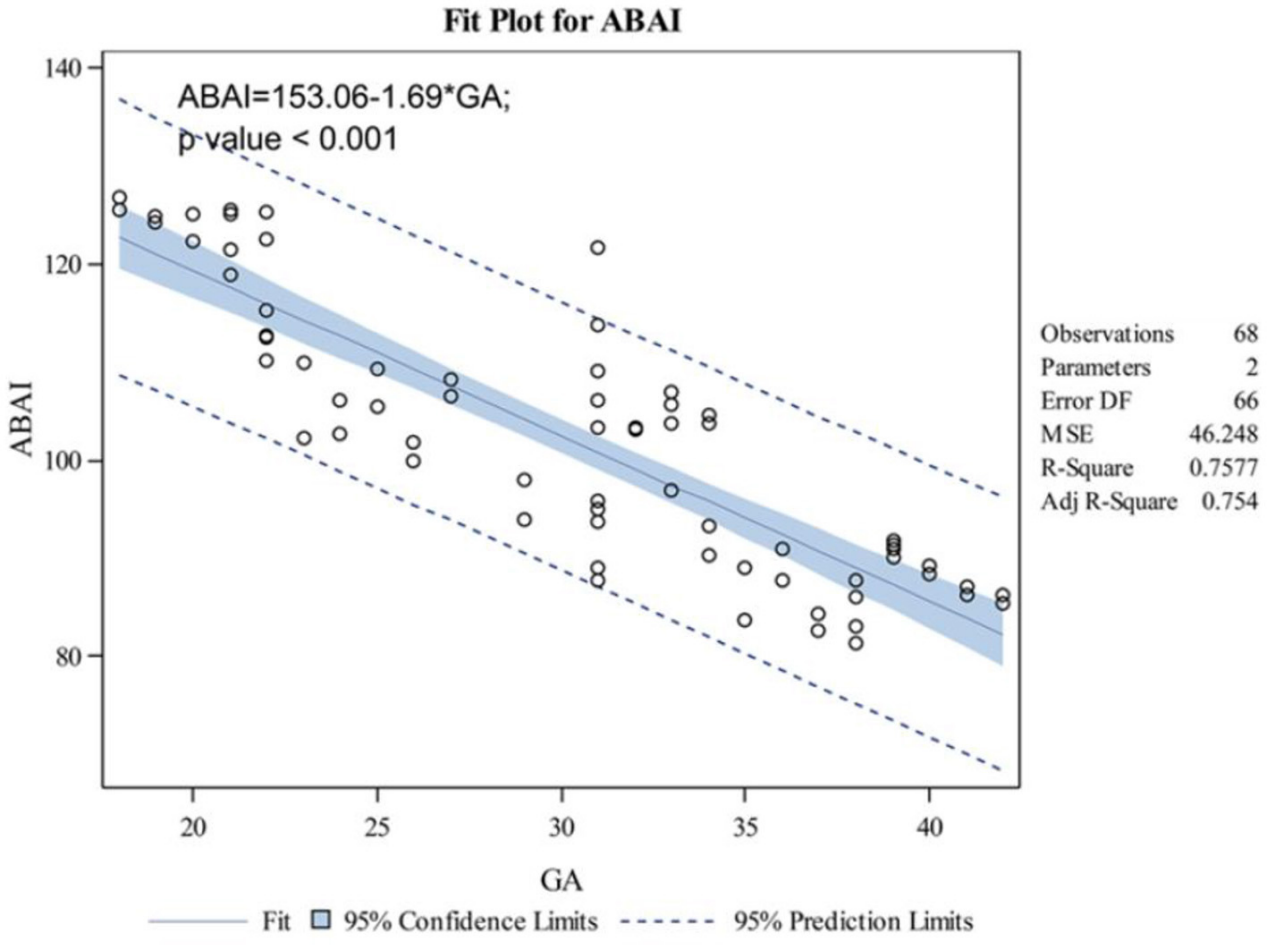
Correlation between gestational age and anterior bony acetabular index.

**Figure 7:**
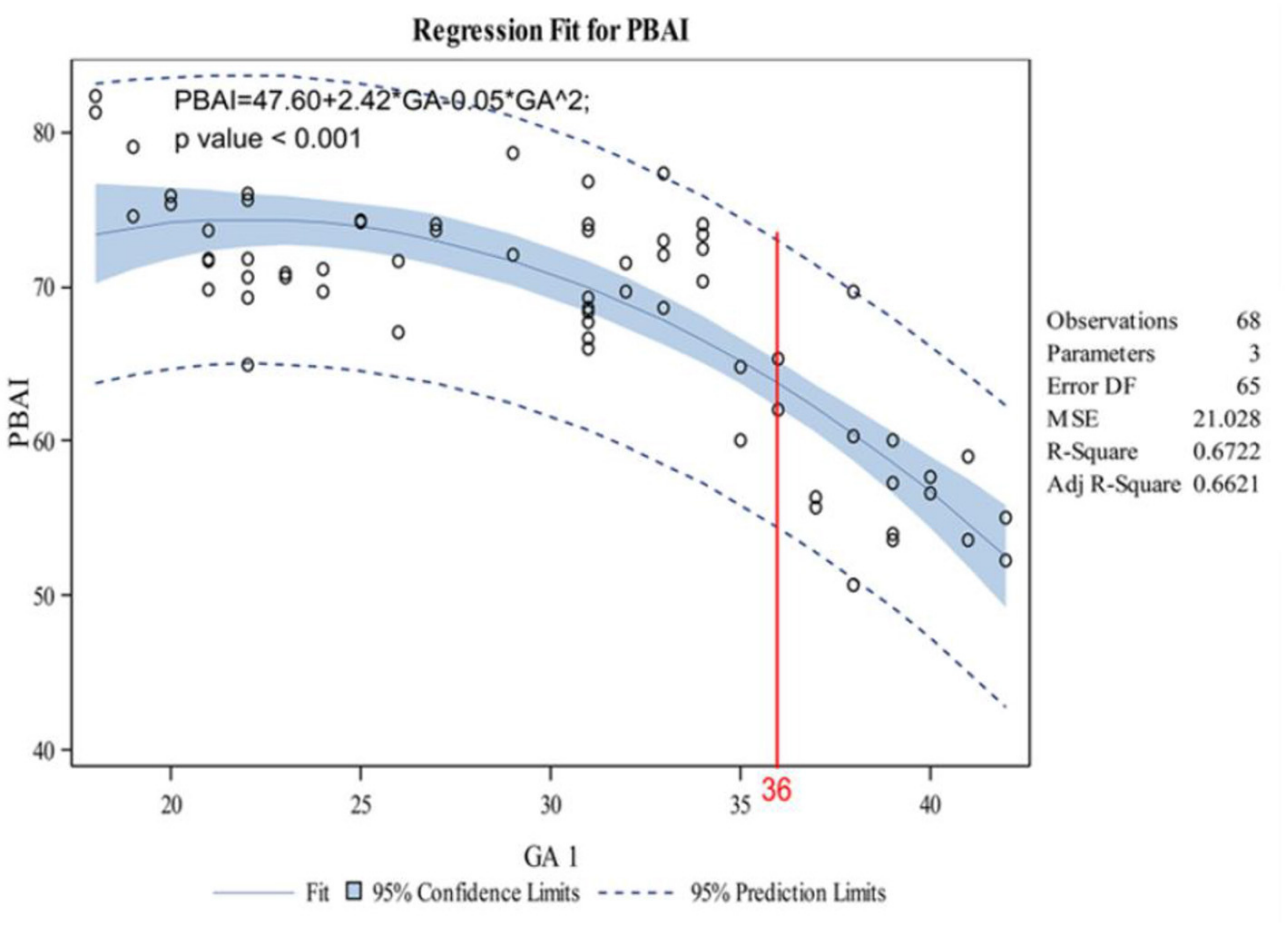
Correlation between gestational age and posterior bony acetabular index.

**Figure 8:**
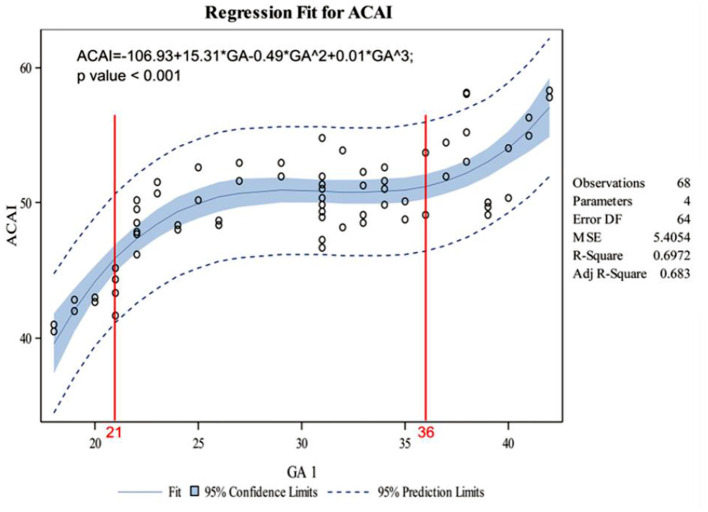
Correlation between gestational age and anterior cartilaginous acetabular index.

**Figure 9:**
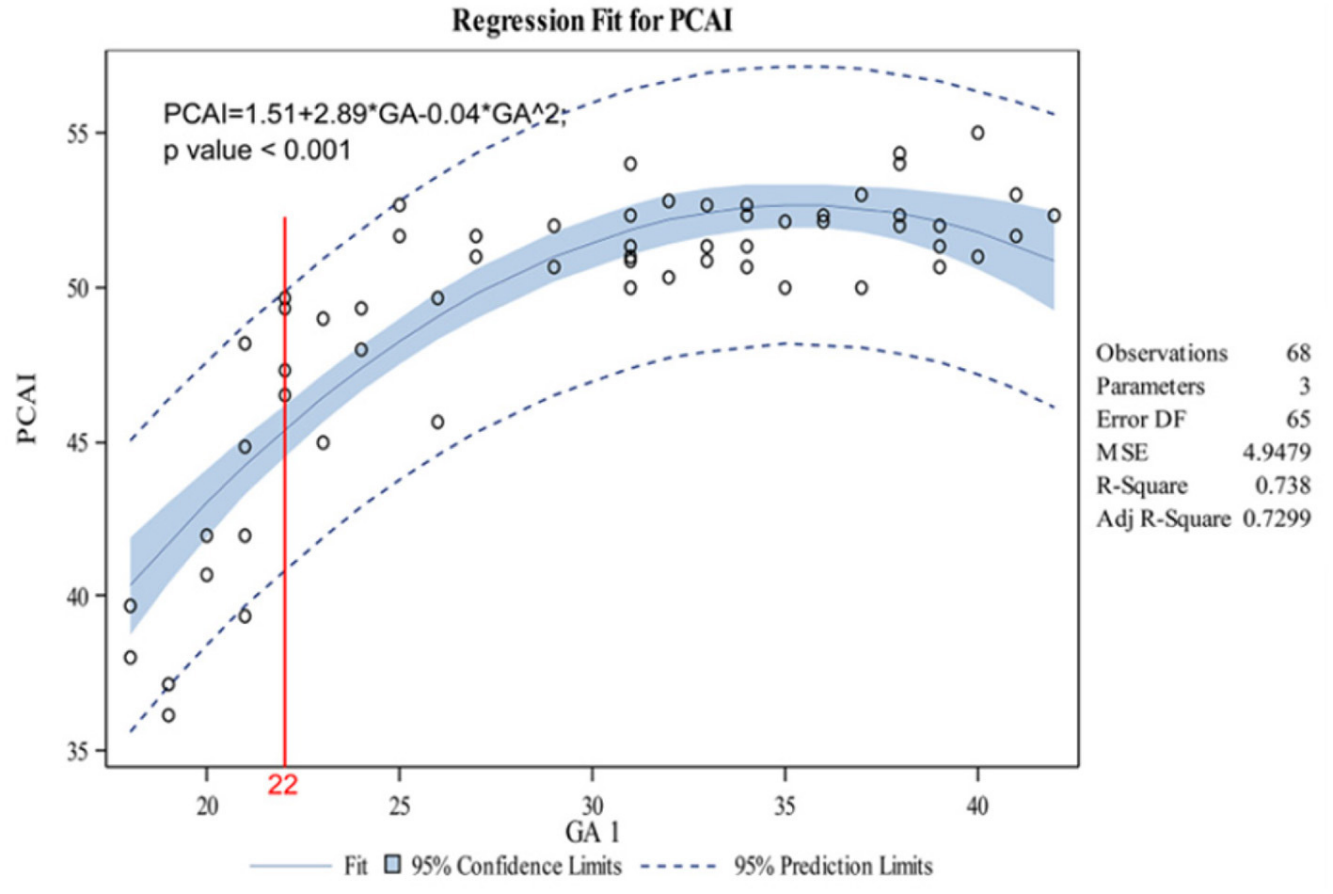
Correlation between gestational age and posterior cartilaginous acetabular index.

The indicators changed at different gestational ages and at different rates. As the gestational age increased, the acetabular width and depth increased linearly. The slope of gestational age against acetabular width was 0.05 (adjusted R2 = 0.86). Two change points in width were detected in the 24th and 34th weeks of gestation. These data represented three segments, and the middle segment decreased at a slower rate compared with the first and third segments (Figure 4). The slope of gestational age against acetabular depth was 0.01 (adjusted R2 = 0.73; Figure 5). Decreases in the ABAI and PBAI were noted during fetal hip development. The ABAI demonstrated an approximate linear trend. The slope of gestational age against the ABAI was −1.69 (adjusted R2 = 0.75; Figure 6). However, a non-linear trend was noted with regard to the PBAI (adjusted R2 = 0.66), and a change point was detected in the 36th week of gestation. These data represented two segments, and the former decreased more slowly compared with the latter (Figure 7). The ACAI and PCAI both exhibited a slow increase, indicating a non-linear trend, and the change points in the ACAI were observed at the 21st and 36th weeks of gestation. The ACAI changed slowly from the 21st to the 36th week of gestation, and then increased rapidly (Figure 8). The change point in the PCAI was observed in the 22nd week of gestation. Then, the PCAI increased relatively slowly (Figure 9).

## Discussion

MRI has greater advantages in studying the morphological changes of fetal acetabular cartilage and the development of femoral head epiphysis by virtue of the characteristics of no radiation, good spatial resolution and good imaging of cartilaginous structures, which can provide a large amount of information about the development of hip joints in the fetal period. To clarify the normal incidence of fetal hip joint is the theoretical basis for the early diagnosis and treatment of fetal DDH, and it is conducive to taking effective interventions to reduce the incidence of DDH during pregnancy. The trends between the indicators and gestational age comprehensively illustrated the development of the fetal hip. With the ossification of the acetabulum, the bone segment of the anterior and posterior acetabular coverage of the femoral head increases, but the cartilage segment of the anterior and posterior acetabular coverage of the femoral head decreases to a minimum value at term.

The anatomical details of the fetal hip were clear on T2WI MRI for all of the fetuses included in the present study (12). The parameters obtained in cross section are selected for the fetal presentation instead of the ace-tabular index in the coronal section, so it is difficult to obtain standard coronal section to fetal hip joint. In Buckley's study, a line through the anterior aspect of the triradiate cartilage was seen as the baseline; and the deficiency of anterior and posterior acetabulum was proved by DDH (15). The aforementioned baseline required that the bilateral hip joint was revealed in a transverse plane. However, the standard transverse plane was difficult to obtain for the fetal presentation. Generally, the acetabulum and femoral head are round and had the same point (16). Therefore, a line was drawn along the cross section from the thinnest point of the acetabular wall to the center of the femoral head. This line is considered to be the baseline to avoid using the contralateral acetabulum as a reference of calculation. In order to determine the baseline, the influence of the passive fetal position on the measurement parameters of nuclear MRI was avoided, and thus the evaluation of the fetal hip circumference of pregnant fetus was provided. The data represented three segments. Compared with the first and third segments, the middle segment decreased at a slower rate. These data indicated that the coverage of the acetabulum of the femoral head decreases between the 21st and 36th weeks of gestation, and this values decreases rapidly before 21 weeks or after 36 weeks of pregnancy. Based on an ultrasonographic study of the prenatal hip joints by Baróti et al., the most intensive growth of the head occurs at 24 weeks of gestation (8). Whitby et al. demonstrated an exponential increase in acetabular width until 24 weeks of gestation (12). Rális showed that at the 11th week of pregnancy, the spherical femoral head was almost completely surrounded by the deepest acetabulum. Then, the acetabulum became shallower, and the femoral head rapidly increased in size. At birth, the acetabular coverage rate of femoral head before delivery was the lowest (18). As far as PCAI is concerned, the change points was observed in the 22nd week of gestation, and then the value slowly increased. Therefore, after 22 weeks of development, the posterior cartilaginous acetabulum remained relatively stable. Harnroongroj et al. described this baseline on computed tomography and it has been demonstrated to be a reliable method (16).

As a fetus develops, the ACAI and PCAI exhibit a slow increase, indicating a decrease in the anterior and posterior femoral head coverage of the cartilaginous acetabulum. This conclusion is consistent with previous research studies (1, 8, 12, 18). As far as ACAI is concerned, the change were observed in the 21st and 36th pregnancy. In perinatal period, insufficient anterior coverage and relatively stable posterior coverage of acetabulum might explain why the unstable hip is prone to anteroposterior dislocations at birth (19).

In addition, the changes of width and depth of acetabulum during the development of fetal hip joint were analyzed, and these values increased linearly with the development of the acetabulum. Based on the variation of the acetabular width and depth, the former increased more rapidly compared with the latter, and the acetabulum became shallower as gestational age increased. A previous anatomical study have shown that the acetabular depth was the slowest variable of growing hip, and it has increased <4 fold during the period studied. This study also showed that in the 18th semester, shallow nest (18). In addition, two change in width were detected at the 24th and 34th weeks of pregnancy. These data could be separated into three segments, and the middle segment decreased slowly compared with the first and third segments. These data demonstrate the slower growth of the acetabulum during the second trimester, and the acetabulum developed rapidly after 34 weeks of pregnancy. The rapid growth of the acetabular width and the slower growth of the depth led to a less stable hip during perinatal life (20). Early diagnosis and good prognosis of DDH disease need to pay more attention to the development of fetal hip joints in perinatal period.

The ABAI and PBAI decreased, which indicated that the acetabular coverage of anterior and posterior femoral head increased with acetabulum ossification. However, an ultrasound study found that with the development of fetus, the α angle gradually decreased (18). The positive correlation between femoral head coverage and the α angle was confirmed (21). The conclusions of this study are different from those of these ultrasonography. This difference was likely because of the disparate indicators measured. The acetabular coverage of the femoral head was divided into four parts: anterior, posterior, medial and lateral (22). The ultrasound revealed the α angle formed between the baseline (parallel to the lateral iliac border) and the crest line (tangential to the bony acetabular roof) along the coronal plane of hip joint (8). The α angle represented the superior bony acetabular coverage of the femur head. In this study, these parameters were measured along the transverse plane, indicating the anterior or posterior coverage of acetabulum of femoral head. Moreover, the baseline used in the present study is likely to influence the angles. This issue needs further discussion. The change point of PBAI was observed in the 36th week of gestation, and the changed was slow before 36 weeks of pregnancy. These data indicated that before the 36th week of pregnancy, the ossification of posterior acetabulum is relatively slow.

Although this study intended to alert clinicians to the early detection of abnormal prenatal hip development by providing normative femoral head coverage parameters, the relatively small sample sizes limited the generalizability of these values. The sample sizes of the different age classes were small, and the reference values of normative femoral head coverage were restricted. More patients need to be further studied to provide an accurate database for measuring development of the hip joint. In addition, there may be some differences between the fetal ages of cadaveric fetuses and the actual fetal age. This study used cadaver fetus data to draw the normative curve. The conclusion of this study should be inferred carefully. A study on *in vivo* fetal imaging is needed to confirm these data before introducing fetal hip MRI in the clinical practice. Moreover, the measurement described in this study avoids the influence of the passive fetal position, and can evaluate the coverage of femoral head of the fetal hip joint in uterus. However, the MRI data of the intrauterine fetal hip were not included in this study. Therefore, the MRI data of the fetal hip joints during intrauterine life should be used to verify the sensitivity and specificity of these indicators.

In conclusion, these preliminary findings illustrated the development of the fetal hip joint in the second and third trimesters. As the development of fetus, the coverage of anterior and posterior cartilage acetabulum of the femoral head continues to decrease to a minimum value at full term, while the coverage of anterior and posterior bony acetabulum of the femoral head keeps increasing. As a normative reference, these data have the potential to alert clinicians to the early detection of abnormal hip development during intrauterine life.

## Data Availability Statement

The original contributions presented in the study are included in the article/supplementary material, further inquiries can be directed to the corresponding author.

## Ethics Statement

The studies involving human participants were reviewed and approved by the Ethics Committee of Guangzhou Woman and Children's Medical Center. Written informed consent to participate in this study was provided by the participants' legal guardian/next of kin. Written informed consent was obtained from the individual(s) and minor(s)' legal guardian/next of kin, for the publication of any potentially identifiable images or data included in this article.

## Author Contributions

ZL is responsible for writing the paper. HLi is responsible for the collection of cases. SW is responsible for the evaluation of the results. QW is responsible for data statistics. HLiu is the instructor of the entire study. All authors contributed to the article and approved the submitted version.

## Funding

This study was funded by the General project of Natural Science Foundation of Guangdong Province (2020A1515010164).

## Conflict of Interest

The authors declare that the research was conducted in the absence of any commercial or financial relationships that could be construed as a potential conflict of interest.

## Publisher's Note

All claims expressed in this article are solely those of the authors and do not necessarily represent those of their affiliated organizations, or those of the publisher, the editors and the reviewers. Any product that may be evaluated in this article, or claim that may be made by its manufacturer, is not guaranteed or endorsed by the publisher.
